# A Braille Trail for all: Inclusive design in the Karoo Desert National Botanical Garden

**DOI:** 10.4102/ajod.v14i0.1764

**Published:** 2025-10-17

**Authors:** Susanna F. Greyling, Suna M. Verhoef, Wilhelm G.d.V. Tempelhoff

**Affiliations:** 1 Research Unit Languages and Literature in the South African Context, Faculty of Humanities, North-West University, Potchefstroom, South Africa; 2 Pioneer School for the Visually Impaired, Worcester, South Africa; 3 Die Virtuele Instituut vir Afrikaans, Pretoria, South Africa

**Keywords:** Braille Trail, accessibility, universal design, sensory design, accessible tourism, information technology, site-specific, locative literature, visually impaired, botanical garden, sensory gardens

## Abstract

**Background:**

Disability-inclusive public green spaces are vital for universal accessibility and for enhancing accessible tourism. Integrating multisensory stimuli with information and communication technologies fosters inclusive, sustainable, interactive, and site-specific tourism experiences.

**Objectives:**

The objective of this article is to present the development of the Braille Trail in the Karoo Desert National Botanical Garden (KDNBG), South Africa, highlighting how participatory design, inclusive multisensory gardens and locative literature foster accessible tourism experiences, while addressing gaps in the literature on sensory and wellbeing gardens from a Global South perspective.

**Method:**

A qualitative, practice-based, and participatory approach was adopted, grounded in principles of collaborative, community-based research. Semi-structured interviews, guided by a thematic framework, elicited insights from participants directly involved in the project. The authors’ practice-based contributions complemented these findings.

**Results:**

The design and establishment of the Braille Trail involved collaboration between Garden management and staff, visually impaired persons, service organisations, institutional partners, and creative contributors. This inclusive process ensured that the trail reflected the needs, experiences, and expectations of its intended users.

**Conclusion:**

The Braille Trail integrates sensory garden design, accessibility, and diverse communication technologies – including digital platforms and locative literature – while incorporating indigenous elements to enrich visitor experiences. Continued community engagement, together with lessons drawn from successes and challenges, provides guidance for sustaining and extending inclusive design in future projects.

**Contribution:**

This study offers insight into multisensory gardens and accessible tourism in a Global South context, demonstrating the application of universal and inclusive design, co-design, slow tourism, accessible communication technologies, and collaborative approaches to create engaging and accessible visitor experiences.

## Introduction

Over the past century, society has increasingly recognised the positive impact of access to green outdoor spaces, such as gardens, on wellbeing. Research shows that gardens can enhance physical health, physical mobility, mental development and emotional growth (De Bell et al. 2020; Gill 2014; Hussein, Abidin & Omar 2013; Hussein, Omar & Ishak 2016). Horticultural therapy emerged early as a field that emphasised the therapeutic value of nature through the development of various garden types – such as wellbeing, learning and multisensory gardens – in settings such as hospitals, rehabilitation centres and special schools for disabled learners (Davis 1998; Hauk et al. 2018; Shoemaker 2014; Zajadacz & Lubarska 2020). The concept of creating multisensory gardens specifically for persons with visual impairment (VI) is well-established (Hussein et al. 2013).

More recently, however, multisensory gardens have moved beyond exclusively therapeutical and educational settings into more inclusive and public environments, such as parks and botanical gardens (Zajadacz & Lubarska 2020). This shift reflects a broader global emphasis on moving away from secluded, specialised spaces towards accessible public environments for persons with disabilities. A key driver of this shift has been the rise of international agendas promoting disability-inclusive development and the rights of persons with disabilities.

In September 2015, the 193 member states of the United Nations adopted the 2030 Agenda, an integrated sustainable progress and development framework (Happ & Bolla 2022). Goal 11.7 of the Agenda specifically states that by 2030 there should be universal access to safe, inclusive and accessible, green and public spaces, particularly for women and children, older persons and persons with disabilities (Brolan 2016; United Nations 2015). The 2030 Agenda incrementally builds on the international community’s development and adoption of the United Nations Convention on the Rights of Persons with Disabilities (United Nations 2011). In Article 30 (United Nations 2011), member states commit to ensuring that persons with disabilities have access to sporting, recreational and tourism venues.

By integrating multisensory gardens into public parks and botanical gardens, these spaces can become potential tourism destinations aligned with the principles of accessible tourism. Buhalis and Darcy (2011) define accessible tourism as:

[*A*] form of tourism that involves collaborative processes between stakeholders that enables people with access requirements, including mobility, vision, hearing and cognitive dimensions of access, to function independently and with equity and dignity through the delivery of universally designed tourism products, services and environments. (p. 10)

This definition reflects a whole-of-life approach, highlighting that accessible tourism benefits people of all ages and circumstances. These include persons with permanent or temporary disabilities, older adults, persons with obesity, families with young children, and those working in environments designed to be safer and more socially sustainable (Buhalis & Darcy 2011:10).

Accessible tourism is increasingly no longer viewed as a niche concern, as illustrated by the United Nations World Tourism Organization designation of ‘Accessible Tourism for All – Promoting Universal Accessibility’ as the theme for the International Day of Tourism in 2016 (Ambrose 2022). In South Africa, this global emphasis has been echoed in national efforts: the Department of Tourism hosted a Public Lecture forum in 2016, where Therina Wentzel, National Director of the National Council for Persons with Physical Disabilities in South Africa, delivered the keynote address. Her presentation focused on strategies to enhance and promote universal accessibility in the South African tourism sector (Department of Tourism 2016).

As accessible tourism is a relatively new field of study, relevant frameworks are still being developed. Happ and Bolla (2022) propose a new sustainability model that integrates perspectives from tourism, disability studies and special needs education. Their model highlights three key priorities: preparing the tourism sector to welcome persons with disabilities; ensuring that destinations adopt strategies tailored to the specific needs of this target group and promoting cross-sectoral cooperation. As they argue, ‘[*by*] linking tourism and actors from other functional sectors (e.g. mobility providers) a socially sustainable offer can be developed’ (Happ & Bolla 2022:6).

Agapito and Guerreiro (2023) argue for a framework that includes a participatory approach focusing on multisensory stimuli centred on local resources, combined with virtual and augmented environments. This framework can create accessible and sustainable tourism experiences. Agapito and Guerreiro (2023) posit that communication technology can be used to optimise multisensory aspects of unique local resources. The significance of this framework is threefold: it strengthens local identities and aids sustainable tourism by involving the community; it promotes experiences deemed authentic considering multiple stakeholders’ perspectives; and it addresses the visitor experience before, during and after the visit to the destination in an accessible manner using smart technologies.

To support the development of accessible experiences such as multisensory gardens and braille trails, it is important to incorporate the voices and embodied knowledge of persons with disabilities. This approach aligns with the disability rights principle of ‘nothing about us without us’, which emphasises the importance of involving persons with disabilities in research, policy-making and design (Dickson, Darcy & Schweinsberg 2024). One practical way to realise this principle is through co-design, a collaborative method that brings together diverse stakeholders – including community members, service providers and local authorities – to create innovative products and experiences that are beneficial for all (Abat & Bhalla 2022:523). Given that users have diverse accessibility needs, the recommended design approach is to follow the principles of inclusive design (Zajadacz & Lubarska 2020). Universal design is based on the idea that anything being designed should consider as many types of users as possible from the outset, rather than relying on retrofitted adaptations (Darcy & Dickson 2009). Centring lived experiences of local users, in this case, the bodily experiences of disabled persons, on creating interesting multisensory experiences, on safety (but not to the point of making it boring) and on slowing down, ties in with the international movement towards slow tourism. Slow tourism emphasises longer stays and authentic experiences that highlight local nature, culture and heritage (Dickinson 2022). It is closely linked to the idea of place attachment and sustainable tourism development. Braille trails and multisensory gardens in parks, botanical gardens or nature reserves are designed to make outdoor spaces more accessible and enjoyable for persons with (visual) disabilities, enabling them to experience the natural environment more closely, gain knowledge about it and develop a deeper connection with nature. Where botanical gardens are conducive to slow tourism (Titus & Spencer 2015:1241) facilities such as fragrance gardens and braille trails have an additional slowing effect (Lin, Huang & Ho 2020; Özdemir & Çelebi 2018).

The establishment of a universally accessible Braille Trail in the Karoo Desert National Botanical Garden (KDNBG) exemplifies a participatory initiative that integrates multisensory stimuli drawing on local resources with information and communication technologies (ICT) designed to create accessible, sustainable tourism experiences and foster a sense of place.

The Braille Trail itself is a 154-m wheelchair-accessible loop featuring tactile elements and interpretation boards that incorporate braille, text and images. The installation is further enhanced by ICT, enabling visitors to scan quick response (QR) codes with a cellphone or similar device to access audio recordings, multilingual site-specific poems and musical interpretations – enriching the sensory experience for all visitors. A universally accessible braille trail can offer meaningful experiences for the local community while also making a broader contribution to accessible tourism.

The Karoo Desert National Botanical Garden in Worcester, located in the Breede Valley of the Western Cape, South Africa, provides an ideal setting for such a project. As a conservation garden, it spans 11 ha of developed land and 143 ha of natural vegetation, showcasing a wide variety of arid and semi-arid plants (Oliver 2010:115). The garden attracts both local and international visitors, including residents from the town and surrounding areas, and serves as a site for training and research. Worcester is home to a large community of persons with VI and hosts several institutions that support persons with disabilities. These include the Innovation for the Blind (formerly the Institute for the Blind), National Institute for the Deaf, De la Bat School for the Deaf, Langerug School for learners with learning difficulties and Pioneer School for the Visually Impaired (hereafter Pioneer School), which caters to blind, partially sighted, deafblind and multidisabled learners.

This article aims to describe the development and establishment of the Braille Trail, informed by a broader research project exploring the design principles and potential applications of locative literature installations and multisensory gardens. In doing so, it seeks to contribute to strategies for accessible tourism and highlights how South Africa can strengthen universal accessibility, while also documenting the project. At the same time, the article situates the Braille Trail within a wider scholarly context: existing literature on garden design principles that enhance enriched experiences and address specific needs – such as sensory gardens, wellbeing gardens and trails accessible to persons with VI – has largely emerged from the Global North. Although these principles may have universal applicability – as illustrated in our discussion of the Braille Trail, where we draw on Harries et al. (2023) on wellbeing gardens and Zajadacz and Lubarska (2020) on sensory gardens for visually impaired users – it remains essential to consider unique local circumstances and challenges. Research from a Global South perspective is scarce and has primarily focused on school grounds and playgrounds (see Masamery et al. 2023; Moseley & Gibbon 2024; Jordaan & Falk 2025). Furthermore, there is a notable international gap in scholarship on integrating accessible communication technology in sensory gardens (Wang 2024). Directly transferring technology from one context to another (South to North or North to South) without re-contextualisation can be problematic (Rivas Velarde et al. 2021:261). Against this background, the article seeks to address both gaps by contributing a locally grounded perspective from the Global South (see Botha & Ohajunwa 2024:2) and by exploring the role of accessible communication technology in designing multisensory garden experiences.

## Research methods and design

This study contributes to Byderhand (Close at Hand), an ongoing interdisciplinary initiative in the Research Focus Area: Languages and Literature in the South African Context at North-West University. Since 2015, the project has undertaken exploratory research into locative literature (site-specific digital literature) as a form of creative expression. Beyond practice, it examines the interplay of place, literature, digital technology, sensory engagement, inclusivity and collaborative knowledge production (Greyling 2018; Greyling, Verhoef & Tempelhoff 2020; Odendaal 2020). The project adopts a qualitative, practice-based, participatory and exploratory approach (Heron & Reason 1997:275–283), drawing on four interrelated knowledge modalities – experiential, presentational, propositional and practical knowledge – to integrate first-hand experience, creative expression, theory and skills in both research and practice. This approach fosters dynamic interaction among participants and knowledge forms, supporting collaborative, community-focused inquiry (Beacon North East 2011:2; Greyling et al. 2020:1344).

The digital installation in the Braille Trail forms part of the broader Byderhand Pioneer Project, which also included *Garden Verses* and *Pioneer Stories* – initiatives developed for the multisensory garden at the Pioneer School (Greyling et al. 2020). During the project’s conceptualisation, the Byderhand team, together with the Pioneer School and the KDNBG, explored integrating a digital literature initiative, *Karoo Garden Poems*, into the planned Braille Trail. Over several years, site visits and follow-up discussions shaped the design solutions – covering content, format, technologies and interfaces – which were first implemented at the Pioneer School in 2018 and later adapted for the Braille Trail in 2022, with additional applications developed in the interim. These elements were incorporated into the trail’s layout and interpretation boards, producing a cohesive, integrated outcome. The authors contributed expertise in project management, creative writing, special needs education and multimedia design, with particular emphasis on locative literature and interface design.

Building on earlier implementations, the current phase of the Byderhand initiative identifies principles for designing inclusive, context-sensitive, and sustainable multisensory gardens and locative literature projects rooted in local environments. It also develops practical and educational guidelines for recreational and learning contexts. Using a hybrid approach, the study combines conceptual analysis with experiential insights from the authors’ projects, interviews and documentation, including project planning documents, visual material, design processes, creative work and field notes. It directly addresses the question: Which guiding principles support the design of purposeful, sustainable and inclusive multisensory gardens sensitive to local contexts?

The participants interviewed for this study constituted the core decision-making team that directed the planning and implementation of the Braille Trail at the KDNBG. They were selected through purposeful sampling based on inclusion criteria related to their roles and expertise: Werner Voigt (former curator; since June 2019, curator of Kirstenbosch National Botanical Garden); Ricardo Riddles (former head horticulturist and curator); Lize Labuscagne (interpretation officer) and Thabang Makola (former intern; currently head horticulturist and acting curator). A thematic framework guided the interviews, with key themes including participants’ roles, stakeholder dynamics, design principles, project challenges, the use and impact of the Braille Trail, educational potential and advice for similar initiatives. While the protocol focused on these predetermined themes, the semi-structured format allowed for in-depth discussion and the emergence of additional insights.

Semi-structured face-to-face interviews of approximately 30 min each were conducted by Author 2 and audio-recorded with participants’ informed consent, including permission to use their names in this article. Recordings were transcribed by Author 1 and verified by Author 2. Thematic analysis conducted by Author 1 followed Braun and Clarke’s (2006) reflexive process – familiarisation, coding, theme development, reviewing, naming and reporting – and the authors collaboratively discussed the findings to support a fair, well-rounded and nuanced interpretation.

The authors were actively involved in the Braille Trail project as coordinator of the Byderhand Project, multimedia designer and special needs educator. Although not interviewees, their direct involvement provided experiential insights that complement the empirical data. Drawing on their scholarly experience, the authors engaged in ongoing self-reflection on their roles and assumptions, consistent with the participatory inquiry paradigm (Heron & Reason 1997). In line with participatory research principles, a draft of the article was shared with a key contributor for fact-checking, ensuring fair representation and ethical co-construction of knowledge (Birt et al. 2016).

In addition to thematic analysis and reflective insights, the evaluation of the Braille Trail was guided by established sensory and wellbeing garden guidelines, along with universal design and accessibility principles, providing a framework to interpret the data and link empirical findings with practical design outcomes.

### Ethical considerations

Ethical clearance for the study was granted on 02 August 2024 by the North-West University Senate Committee for Research Ethics (NWU-SCRE), following approval by the Ethics Committee for Language Matters (ECLM) (reference number: NWU-01104-24-A7).

## Results

### Multidisciplinary perspectives on the Braille Trail’s design and implementation

Thematic analysis of the interviews identified key themes central to inclusive design in the Braille Trail, including collaborative approaches, strategic planning, project management, horticultural and sensory design, and accessible interpretive content. Results are presented according to the trail’s ‘layers’, highlighting design challenges and consideration of local features, alongside solutions implemented in consultation with stakeholders. The subsections on digital enhancements and universal interface design are presented within this layered framework, drawing on the authors’ practice-based contributions to complement the interview findings.

### Collaborative approaches: Multi-stakeholder involvement

The Braille Trail’s planning and development was a multi-year, collaborative process led by the implementation team of the KDNBG and involving institutional partners, persons with VI, service organisations and creative contributors. While delays arose from challenges such as limited funding, procurement issues, staff turnover and the coronavirus disease 2019 (COVID-19) pandemic, the extended timeline also allowed for deeper reflection and broader participation (R. Riddles, pers. comm., 20 February 2025; W. Voigt, pers. comm., 11 March 2025). This inclusive, iterative approach ensured the trail’s design responded to the diverse needs and experiences of its users.

Overall project oversight rested with the Director of National Botanical Gardens, whose approval was essential for implementation. The renewal and enhancement of the Braille Trail aligned with the South African National Biodiversity Institute (SANBI) annual performance plan targets, embedding the project within broader institutional goals (W. Voigt, pers. comm., 11 March 2025).

Staff at the KDNBG played a central role throughout the project cycle, from conceptualisation to implementation and ongoing maintenance. Key contributors included the curator, horticulturist, interpretation officer and gardening staff (R. Riddles, pers. comm., 20 February 2025; W. Voigt, pers. comm., 11 March 2025).

South African National Biodiversity Institute’s Education Division contributed to the development of interpretive content and educational signage. The upgraded trail further supports their ongoing outreach programmes, including regular site visits by school groups (W. Voigt, pers. comm., 11 March 2025).

Key external partners included North-West University’s Byderhand Project and the Pioneer School. These collaborators were instrumental in the co-design process, particularly in integrating ICT and ensuring the trail addressed the needs of visually impaired users (W. Voigt, pers. comm., 11 March 2025).

The primary users and beneficiaries are persons with VI – especially learners from the Pioneer School – as well as the wider community (W. Voigt, pers. comm., 11 March 2025). Their active participation, especially in user testing and interface design, was vital to the project’s success.

Additional contributions came from organisations working with visually impaired persons, including Innovation for the Blind and Pioneer Printers. Creative collaborators – poets and musicians – contributed to the Karoo Garden Poems, while funders such as the Botanical Society of South Africa (BotSoc), the Cape Winelands District Municipality, and the Rowland and Leta Hill Trust provided essential financial support (L. Labuscagne pers. comm., 26 November 2024). Tourism bodies such as Worcester Tourism further supported the project through facilitation and marketing (L. Labuscagne pers. comm., 26 November 2024).

### Strategic planning and design principles

The new trail replaced an older version that had largely fallen into disuse. Issues with the previous route directly informed the design of the new one. Most challenges were practical and hindered navigation, especially for persons with VI. Key problems included poorly marked start and end points, deteriorated signage, non-compliant plants and uneven surfaces such as shale, gravel and lawn, making access particularly difficult for visually impaired users and wheelchair users (L. Labuscagne pers. comm., 26 November 2024; R. Riddles, pers. comm., 20 February 2025; W. Voigt, pers. comm., 11 March 2025).

In planning the new Braille Trail, W. Voigt (pers. comm., 11 March 2025), then curator of the KDNBG, focused on expanding interpretive content and increasing the trail’s accessibility and appeal to a broader audience. W. Voigt (pers. comm., 11 March 2025) outlined several considerations that guided the planning and implementation:

- **Accessibility:** A primary consideration was ensuring that the trail could be navigated with ease by all users, placing inclusive and user-friendly design at the centre of the project.
- **Audience and context:** Adapting content and format to engage both children and adults.
- **Cost:** Managing budgetary constraints, especially in material choices and digital integration.
- **Materials:** Selecting durable, sustainable and accessible materials for paths and signage.
- **Technological integration:** Exploring the incorporation of smartphone-compatible digital tools to enhance access and experience.
- **Trail length and layout:** Designing a compact, easy-to-navigate route suitable for all visitors.
- **Themes and plant selection:** The focus broadened to include culturally and socially significant plants, while maintaining an emphasis on Karoo-adapted species.

### Project management and site selection

Riddles, the horticulturist responsible for managing the project (R. Riddles, pers. comm., 20 February 2025), oversaw tasks including gathering information on the project scope, planning the trail and its layout, and selecting suitable plants.

During the planning of the new trail, an initial location was identified, and the planning and budgeting process commenced. However, it gradually became evident that this site was unsuitable for the proposed route. The path would have followed a slope that was overly steep, with inclines and declines rendering it inaccessible to wheelchair users. Budgetary constraints also presented challenges. Everlasting decking – a durable, wood-like plastic material – was considered for its longevity, but its high cost raised concerns. In addition, the terrain required the construction of a small bridge, further increasing the overall expense. It was subsequently decided to develop the trail alongside the new Environmental Education Centre, which was under construction. This terrain – a relatively level area of natural vegetation – simplified the layout and construction of the path, significantly improved accessibility, helped to reduce costs and allowed for greater creativity (R. Riddles, pers. comm., 20 February 2025).

As part of the project planning, Riddles conducted research by visiting various local braille trails and facilities and familiarised himself with international standards. Experts were also consulted, including Innovation for the Blind and the orientation and mobility specialists at the Pioneer School (R. Riddles, pers. comm., 20 February 2025).

### Horticultural design and sensory landscaping

Thabang Makola, a SANBI-funded postgraduate student at the KDNBG, designed the trail as part of his horticultural duties (T. Makola, pers. comm., 12 March 2025; R. Riddles, pers. comm., 20 February 2025). The project was proposed by his then-supervisor, Riddles, who envisioned a braille trail accessible to users with disabilities. Makola approached the task as a commissioned design, with a brief to base it on a native organism of the Succulent Karoo – preferably one with a recognisable overhead shape. After considering various options, the endemic Dwarf Karoo Girdled Lizard (*Cordylus minor*) was selected as the design model.

The layout was designed with consideration of the location, vegetation and biodiversity of both the specific garden section and the larger KDNBG. Trail ‘stations’ were identified in collaboration with the implementation team to highlight key features of the Karoo Garden and its surroundings (L. Labuscagne pers. comm., 26 November 2024).

The existing vegetation provided a natural setting, while selected plants reinforced thematic or functional aspects of the trail, especially in raised beds. Plant choices were guided by the local flora and aimed at multisensory engagement through texture, scent and taste (R. Riddles, pers. comm., 20 February 2025). Special attention was given to plants of educational value for users with VI, including those with tactile qualities, strong fragrances or bird-attracting properties (T. Makola, pers. comm., 12 March 2025). The tactile experience was further enhanced by an exhibit of regional rock types, highlighting the geology that shapes the Karoo vegetation. Given the high cost of composite decking, paving stones were selected as a cost-effective alternative for the pathway (T. Makola, pers. comm., 12 March 2025).

Community consultation informed key planning decisions. An orientation and mobility specialist from the Pioneer School recommended a paving edge – rather than a guide rope – to support navigation with white canes. This decision also addressed hygiene concerns during the COVID-19 pandemic by reducing contact with shared surfaces (R. Riddles, pers. comm., 20 February 2025).

After site clearing, hard landscaping began (T. Makola, pers. comm., 12 March 2025). An external contractor laid the paving, while garden staff performed raised bed construction, relocation of rocks, planting, installation of interpretation boards and related tasks (R. Riddles, pers. comm., 20 February 2025). Yellow-painted tactile pavers were installed to mark points of interest along the route.

### Developing accessible interpretive content

The environmental interpreter, Lize Labuscagne, led the development of the interpretation content, beginning with the selection of themes in consultation with the implementation team. The goal was to introduce visitors to key aspects of the Karoo in an accessible and engaging manner (L. Labuscagne pers. comm., 26 November 2024).

Usability was a central consideration, ensuring content that visitors would actively engage with and enjoy. As most visitors are sighted, the interpretation boards also needed to be visually appealing and attention-grabbing. The goal was to tell a compelling story, not just present facts, fostering a sense of connection with the content (L. Labuscagne pers. comm., 26 November 2024). Given the wealth of available information, the content had to be carefully curated for relevance and clarity (L. Labuscagne pers. comm., 26 November 2024; W. Voigt, pers. comm., 11 March 2025). A key guideline was to write at a comprehension level suitable for a 12-year-old: clear, concise and engaging (L. Labuscagne pers. comm., 26 November 2024).

After finalising the themes and design, the implementation team consulted Innovation for the Blind – a Worcester-based non-profit organisation that empowers visually impaired adults and older persons through training, development and specialised care – to further enhance accessibility (L. Labuscagne pers. comm., 26 November 2024). The final boards feature a background photo and a bilingual (English and Afrikaans) description, reverse-printed on dibond for durability (W. Voigt, pers. comm., 11 March 2025). Each board includes a bilingual braille panel produced by Pioneer Printers – a Worcester-based non-profit organisation that produces braille, large print and audio formats – as well as a braille-framed QR code linking to digital content.

In addition to seven interpretation boards, three orientation boards were planned to guide visitors: an entrance board to present the trail layout, a second to explain the use of QR codes and a final board at the exit to mark the trail’s conclusion.

### Digital enhancements: Site-specific poems and interactive media

This section draws on the authors’ practice-based contributions, complementing the interview findings.

The digital installations along the trail route include 10 site-specific poems – referred to as *Karootuinverse* or *Karoo Garden Poems* – alongside a functional component: the provision of information in digital format. These installations were developed and implemented by the Byderhand Project from North-West University, which focuses on the creation and digital installation of site-specific literature.

Site-specific literature refers to literary works created in response to, and in meaningful connection to, a particular location. These works often draw on the site’s history, geography, culture or sensory qualities, aiming to enhance the visitor’s experience through storytelling, poetry and other narrative forms (Farman 2014:3; Greyling 2018:208). Presented in a digital format, the Byderhand Project creates layered experiences by combining text, sound, image and video in different ways across contexts. The works are hosted on the Byderhand digital platform (www.byderhand.net) and accessed via QR codes and a dedicated interface.

A distinctive feature of the Byderhand model is the involvement of local writers in content creation. For the Karoo Garden project, poets with ties to Worcester and the surrounding areas were invited to contribute poems that reflect the garden environment, Karoo fauna and flora, or the landscapes of the Breede and Hex River valleys (Odendaal 2020:1369). Contributors included two poets with VI, William Rowland and Jacques Coetzee, together with Diana Ferrus, Pieter Hugo, Daniel Hugo, Suenel Bruwer Holloway, David Kramer, F.W. de Jongh, Floris A. Brown and Basie Duvenhage. The 10 poems featured in the installation were originally written in Afrikaans. For the Karoo Garden installation, seven were translated into English, two into isiXhosa, five into Portuguese and six into German. The original poems were read aloud by the poets themselves, while fluent speakers recorded the translations. Some also feature musical adaptations contributed by the poets (Van der Merwe 2020:109). Each poem is accompanied by a short poet biography on the interface. Visitors can read or listen to the poems via the digital interface while exploring the natural surroundings.

### Universal interface design for accessibility

This section draws on the authors’ practice-based contributions, complementing the interview findings.

In response to the invitation to contribute site-specific literary installations for the Byderhand Pioneer Project at the Pioneer School and the Braille Trail in the KDNBG, the Byderhand platform faced two primary accessibility-related design challenges: enabling users to access locative literature at specific physical locations using their own devices and ensuring intuitive interaction with the graphical user interface.

The latest version of the platform – developed in collaboration with staff from the Innovation for the Blind’s technology centre and Pioneer Printers – embraces a multilayered, multisensory design, integrating tactile, auditory and visual modes of interaction (Greyling et al. 2020:1354).

Within the field of interaction design, the interface facilitates interaction between humans and computers by means of sensory processes (Galitz 2007:8). Effective human-computer interaction requires an approach that places the user’s goals and expectations as well as cognitive and physical abilities at the core of the interface design process (Galitz 2007:8). Mainstream interface design typically relies on visual elements such as simplified icons or illustrations, further reinforced by the widespread use of touchscreen technology. Although often considered tactile, touchscreens still require visual input to operate effectively. This dependence on sight not only marginalises users in the Byderhand Pioneer context but also risks further excluding a group already underserved by digital design. In response, the project adopted a user-centred design approach that directly involved users throughout the process, avoiding reliance on designer assumptions. User-centred design empowers users to participate in key decisions, raise usability concerns and suggest solutions grounded in their lived experience (Abras, Maloney-Krichmar & Preece 2004:10).

Philip Crouse and Aydienne Britz, instructors at Innovation for the Blind’s technology centre and themselves visually impaired, played key roles in the interface design process. As accessibility experts, they offered crucial insight into how visually impaired users experience and interact with technology. Through exploratory conversations, prototyping, user testing, iterative refinement and collaborative evaluation, the design team deepened their understanding of user needs and developed practical solutions to enhance accessibility (Greyling et al. 2020:1355).

Testing revealed that diverse interaction modes were required to accommodate users differing in vision and confidence levels. For instance, a partially sighted user might benefit from high contrast and large fonts, while a user with very low vision might rely entirely on audio. Cognitive differences, emotional factors and varying levels of technological proficiency further highlighted the diversity of needs. Because universal design principles were embedded from the start, the team was well-positioned to build an inclusive and adaptive platform (Carr et al. 2013:2).

Elzaan Hendriksz from Pioneer Printers played a key role in the design process, bringing extensive expertise in braille and accessible signage. Her collaboration – together with input from both sighted and visually impaired staff at Pioneer Printers – was crucial in developing tactile and print-based solutions for accessing the locative literature.

In both cases, collaboration with community stakeholders through co-design, testing and group discussions provided essential insights that guided the design process and shaped the final product: a multilayered, accessible interface.

The interface was designed as a sequence of layered interactions:

- Tactile interface and access point: The project uses QR codes to grant users access to locative literature on their mobile phones. Because QR codes must be scanned with a device’s camera, they typically require visual interaction. To make this critical step more accessible, each QR code is framed with braille, and clear instructions are provided on how to scan it. QR codes are positioned so that when a user places their phone flat on the signage, the camera aligns with the code. Users are instructed to slowly lift the phone until the QR code is scanned, confirmed by an audio cue, screen-reader notification or vibration.
- After scanning, users enter the main navigation screen, where literature is available in multiple formats and translations. The interface allows for adjustments in contrast and font size, making it adaptable to different degrees of visual acuity.
- For users reliant on audio interaction, the platform is compatible with screen readers. All interactive elements are tagged using HTML (Hypertext Markup Language) and ARIA (Accessible Rich Internet Applications), ensuring that screen readers provide meaningful context and navigational cues.

This multilayered interface approach was also used to make the content of the interpretation boards accessible to users. The recordings for this component of the project were performed by the in-house recording studio at Pioneer Printers.

Learners from the Pioneer School were actively involved in developing the multilayered interface and became familiar with the technology through their engagement with the Byderhand Pioneer installation in the school’s multisensory garden. They also participated in evaluating the Braille Trail prior to its official launch.

## Discussion

Building on the results, this section reflects on the Braille Trail as an inclusive, integrated trail, showing how its diverse features and layers converge, incorporate indigenous elements and shape potential visitor experiences. Framed by sensory and wellbeing garden guidelines and accessibility principles, the discussion examines successes, challenges and lessons for future inclusive garden projects, organised into three subsections: evaluation of sensory and wellbeing garden features, operational considerations and sustainability, and recognition, reception and use.

### Evaluating the Braille Trail through the lens of sensory and wellbeing garden guidelines

In their systematic review of design recommendations for wellbeing gardens, Harries et al. (2023) identified six key principles: accessibility, wayfinding, spatial organisation, variety in planting, inclusion of cultural artefacts and the fostering of serenity. In addition to these guidelines, it is essential to consider the specific location and climate of the garden, as well as its purpose and intended users. Maintenance and environmental sustainability also play a vital role in ensuring long-term success (Harries et al. 2023:196; Krzeptowska-Moszkowicz, Moszkowicz & Porada 2021:12). These principles are equally relevant to other forms of applied gardens, including sensory gardens and braille trails. According to Zajadacz and Lubarska (2020:29), sensory gardens designed for persons with VI should be purposefully created as coherent, enclosed spaces that are clearly distinct from their surroundings. These gardens should stimulate all the senses, emphasise non-visual experiences, and incorporate elements beyond vegetation to fully engage visitors. The effectiveness of such features depends on a foundational level of accessibility. Safety and ease of orientation are particularly important for persons with VI, for whom comfort and security are essential during garden visits (Zajadacz & Lubarska 2020:29).

In the discussion that follows, these guidelines, together with other relevant design principles, serve as a framework for examining how the Braille Trail seeks to create a safe and welcoming environment through multisensory experience design and inclusive opportunities for exploration.

*Accessibility* involves multiple dimensions, including the garden’s physical location and how easily people of differing abilities can access and navigate it (Harries et al. 2023:189). Closely linked to this is *wayfinding*, the process by which individuals orient themselves and move from one location to another, relying on both information gathering and decision-making (Hunter 2010:1). As wellbeing gardens should be easy to navigate while encouraging a sense of discovery (Harries et al. 2023:191), accessible wayfinding is central to their design. Effective wayfinding enhances safety, confidence and user autonomy (Harries et al. 2023:192). Key features of accessible wayfinding design include visual accessibility, tactile and braille elements, auditory cues, mobility-friendly infrastructure, cognitive accessibility, consistent and predictable layouts, and digital wayfinding tools (Symonds 2025). Zajadacz and Lubarska (2020:32), in their study of sensory gardens for persons with VI, emphasise design elements that support orientation, movement, extended visits and effective communication. Many of these elements – spanning sensory garden design, accessibility and wayfinding – are thoughtfully integrated into the Braille Trail.

**FIGURE 1:**
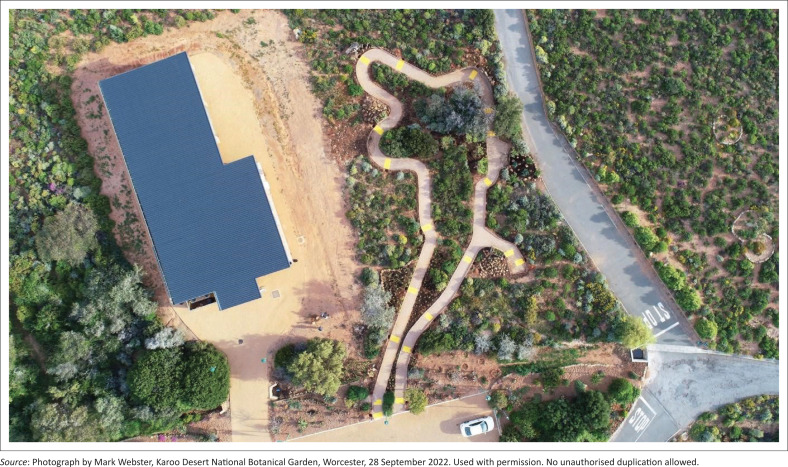
Aerial view of the Braille Trail and environmental education centre in the Karoo Desert National Botanical Garden, Worcester, South Africa.

The Braille Trail, situated in a designated area of the KDNBG, forms a self-contained loop that begins and ends at the same point. Its proximity to the parking area and the Environmental Education Centre enhances overall accessibility (Figure 1).

**FIGURE 2:**
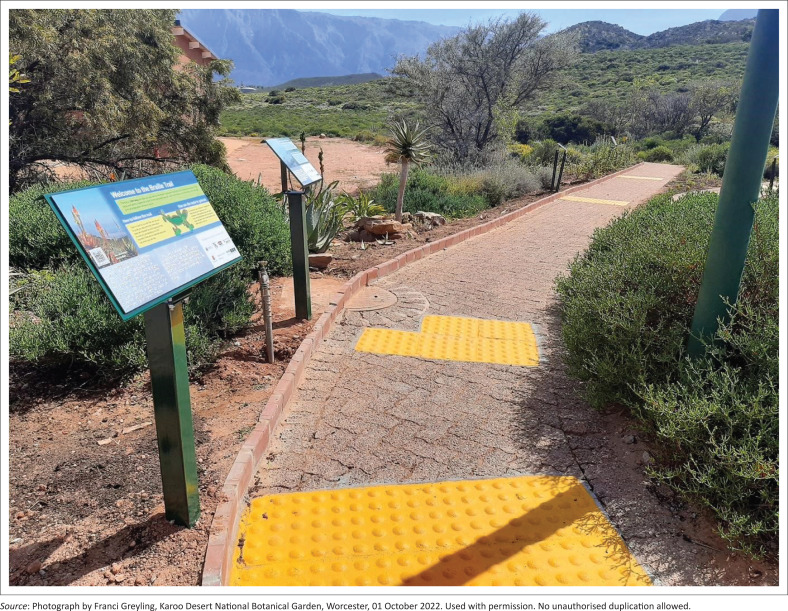
Entrance to the Braille Trail. Spatial orientation is supported through features such as a welcome board with trail information, a clearly defined path layout, a raised kerb, rounded corners, yellow tactile surface indicators and braille-marked waypoints.

Clear paths and accessible signage support orientation and encourage exploration, particularly in wellbeing gardens (Harries et al. 2023). While varied surface textures can enrich sensory engagement, path design should still adhere to universal design principles to ensure accessibility for all users (Zajadacz & Lubarska 2020:30–32). In addition, uniform surfaces improve safety and reduce perceived threat (Harries et al. 2023:190). The Braille Trail supports spatial orientation through a combination of features: a clearly defined path layout, rounded corners, tactile surface indicators, braille-marked waypoints, audio information, mobile applications and optional human assistance (Zajadacz & Lubarska 2020:32). A smooth, wheelchair-friendly surface ensures physical access, while bright yellow tactile paving enhances visibility and helps users locate signage, interpretation stations and site-specific poems (Figure 2). Signage is securely mounted at an accessible height for blind and visually impaired users, while also accommodating wheelchair users (Symonds 2025).

**FIGURE 3:**
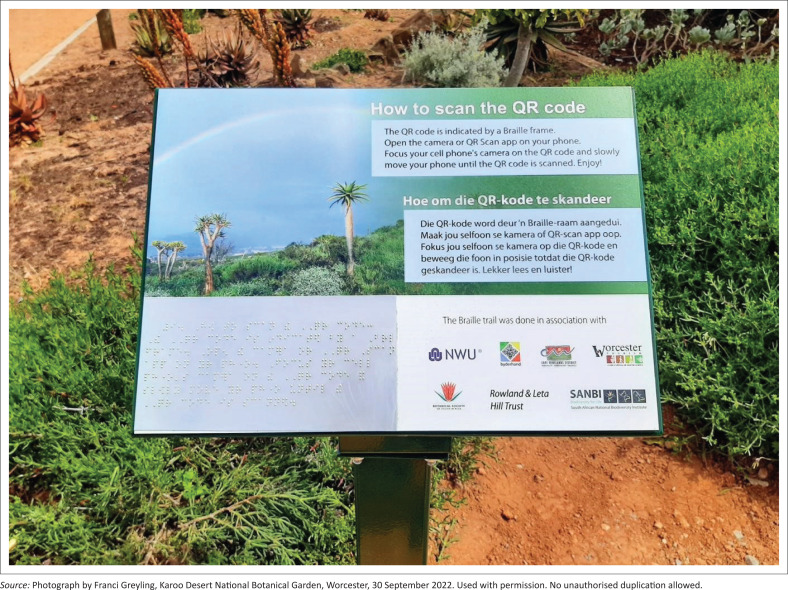
Interpretation board providing instructions on scanning the quick response codes.

Designed to foster independent navigation, the entrance board welcomes and orients visitors to the trail and its features. The Braille Trail incorporates several techniques commonly used in sensory gardens to communicate with persons with VI (Zajadacz & Lubarska 2020:32). Information is conveyed through multiple modes – images, large print, braille and QR codes that link to audio content via an accessible interface. Clear instructions are provided on how to scan the QR codes (Figure 3).

**FIGURE 4:**
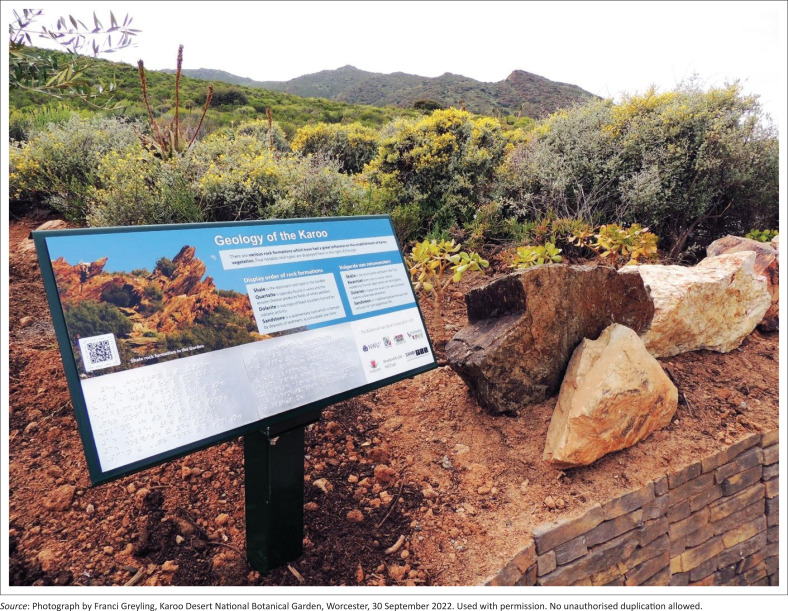
‘Geology of the Karoo’ interpretation board. The clear, engaging language invites interaction through simple activities, such as identifying rock types.

Interpretation enriches visitor experiences by fostering deeper, more mindful engagement, supported by variety, personalisation and visitor agency (Moscardo 2022:777). This approach is reflected in the interpretation boards, which are written at a 12-year-old reading level to support cognitive accessibility (L. Labuscagne pers. comm., 26 November 2024; Symonds 2025). Bilingual text (English and Afrikaans) enhances inclusivity and wayfinding, while digital tools allow for additional content and languages without altering the physical signs. Headings such as *Fascinating Animals of the Karoo* and *Discover Plant Textures* spark curiosity through references to sound, texture and taste. Clear, engaging language invites interaction through simple activities – such as tasting a spekboom (*Portulacaria afra*) leaf or identifying rock types – while the audio guide supports multisensory exploration at one’s own pace (L. Labuscagne pers. comm., 26 November 2024) (Figure 4).

**FIGURE 5:**
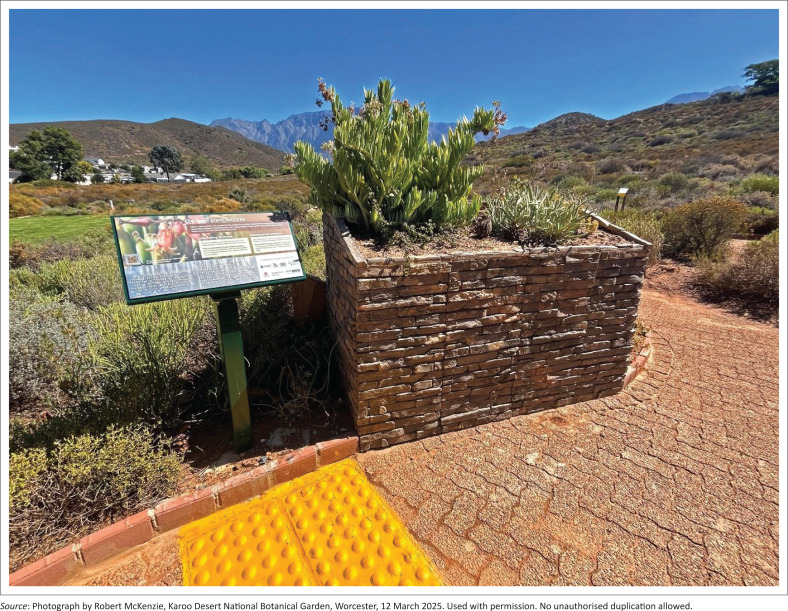
‘Discover plant textures’ station. The use of building stone in the raised beds and constructed walkway contributes to spatial definition and introduces a variety of textures.

*Spatial organisation* in wellbeing gardens should balance the need for social interaction and privacy by including open areas for gatherings and enclosed spaces that offer a sense of safety and seclusion (Harries et al. 2023:195). On the Braille Trail, this balance is supported through the lizard-inspired design (Makoka 2025), which features branching legs that form smaller ‘rooms’ or stopping points. The strategic placement of elevated planting beds and benches within these spaces facilitates both rest and informal social engagement. The use of building stone in the raised beds and constructed walkway contributes to spatial definition and introduces a variety of textures (Figure 5). These elements also enhance mobility support and invite users to pause and interact with their surroundings. Additional wayfinding features, such as curbs, function as tactile navigational aids (Zajadacz & Lubarska 2020:32).

The two benches along the trail provide comfortable resting spots where visitors can easily use their cellphones and engage with the garden poems, either by reading or listening. These areas also promote informal social interaction, supporting shared experiences of the trail and its poetic content. As Harries et al. (2023:191) note, seating placed in both sunny and shaded locations can extend visit duration and encourage use of different parts of a wellbeing garden. While this is a relevant principle, the Braille Trail currently lacks adequate shaded seating. The intense summer heat and arid climate of the Karoo present a challenge in this regard, and, ideally, additional shaded areas should be introduced (R. Riddles, pers. comm., 20 February 2025).

**FIGURE 6:**
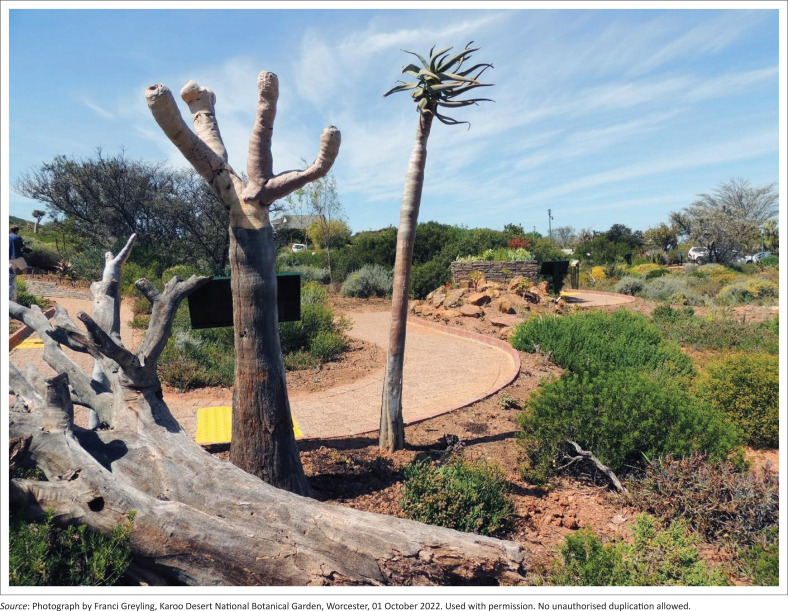
Quiver tree (*Aloidendron dichotomum*) specimens.

A key consideration in the design of sensory and wellbeing gardens is the inclusion of *diverse planting*. These gardens should engage all the senses through thoughtful multisensory planting – incorporating a variety of colours, scents, textures and, where possible, edible plants to create a rich sensory experience (Harries et al. 2023:194; Krzeptowska-Moszkowicz et al. 2021:11). To ensure visitor safety – particularly when encouraging tactile or gustatory interaction – species that are toxic or allergenic should be avoided (Gülgün & Öztürk 2020:167; Harries et al. 2023:194;). The use of indigenous and wild flora can help foster a sense of place and support local biodiversity (Harries et al. 2023:194). In a botanical garden context, the inclusion of varied plantings is central to the sensory experience. The Braille Trail is embedded within the broader landscape of the KDNBG and draws on its abundance of characteristic arid and semi-arid plant species. For the trail’s section on climate change (*The Challenge of Global Warming*), several quiver tree (*Aloidendron dichotomum*) specimens – including both living trees and a fallen dead specimen – have been grouped to create a striking focal point that reinforces the theme (Figure 6).

Gardens with cultural and historical significance can evoke fascination. Fostering a sense of culture within the space can deepen users’ emotional connection, enhance their sense of place and contribute to overall wellbeing (Harries et al. 2023:190). *Cultural artefacts* – such as water fountains, playful features like natural art or sculptures, and interactive devices – contribute to placemaking by offering multisensory experiences that invite engagement, spark curiosity and encourage personal interpretation. These elements deepen the visitor’s connection to the space (Harries et al. 2023:190; Krzeptowska-Moszkowicz et al. 2021:12).

**FIGURE 7:**
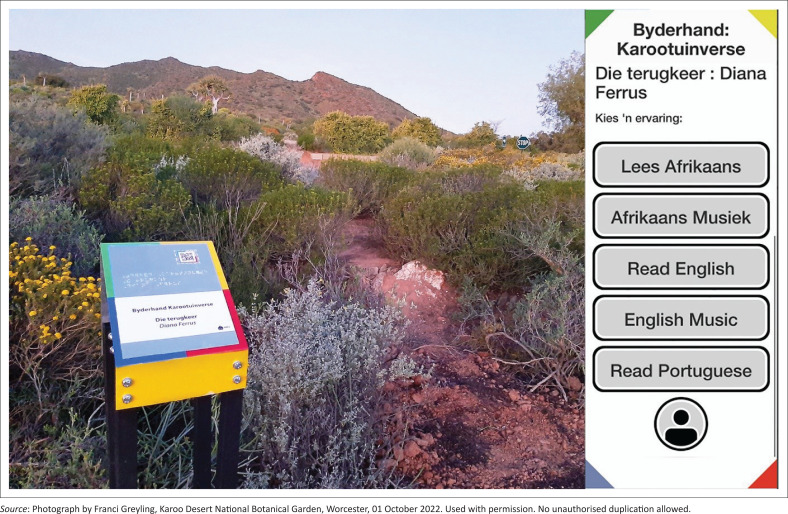
The pedestal and digital interface of the poem, *Die terugkeer* [*The Return*], by Diana Ferrus.

In this context, the poems featured along the Braille Trail can also be understood as cultural artefacts. Presented both as written texts in digital format and audio recordings, the Karoo Garden Poems present 10 unique ‘stories’ or fictional worlds that express a deep appreciation for the distinctive character of the region (Van der Merwe 2020:108). In her poem, *The Return*, Diana Ferrus evokes the landscape through sensory and spatial references – sand paths, mountains, fauna and flora, dry air, and the expansive blue sky of the Breede Valley (Figure 7). Many readers may identify with the speaker’s reflections in the following excerpt:

*Then you walked up the sandpath*
*stumbled across a stone*
*and fell into a thorny bush*
*you forgot the cactus’s name*
*The mountain slopes hung over the valley*,
*the thorn tree, the flowering aloe*
*glittered red in that sun –*
*the one you knew*
*The dry air filled your lungs*
*and the blue expanse charmed you like in earlier times –*
*Nowhere in the world there was such a sky*

The digital elements contribute to an augmented reality experience, adding an immersive, narrative-rich layer to the visitor’s engagement with the place. Because site-specific literature relies on the participant’s physical presence, direct, sensory experience gains renewed significance. The digital component deepens this engagement by encouraging heightened awareness, active participation and meaningful interpretation. As the reader’s attention shifts between physical place, sensory and bodily experience, and digital presentation, the encounter invites a slowing down of time and a deepened sense of presence (Greyling 2018:155).

*Fostering serenity* is also an important quality for restorative garden design. One means to create a serene space is with sound (Harries et al. 2023:192). The recordings of poems in different languages, together with musical adaptations, contribute to a subtle, ephemeral soundscape as visitors listen – encouraging imaginative engagement, fostering a meditative atmosphere and inviting visitors to linger. Natural sounds – such as running water, rustling grasses and birdsong – can further enhance the soundscape (Harries et al. 2023:192) and offer additional opportunities for integration along the Braille Trail. A cool, shaded seating area, accompanied by a sign inviting visitors to focus on bird and animal sounds, could further enrich the sensory experience (R. Riddles, pers. comm., 20 February 2025).

The Braille Trail successfully integrates principles of universal design and sensory garden design to create an inclusive, safe space that welcomes visitors of all ages and circumstances. Its carefully curated features (Botsoc National 2022) combine to offer engaging multisensory experiences that evoke a poetic encounter with the unique landscape.

### Operational considerations and sustainability for long-term impact

Beyond enhancing user experience and sensory engagement, the trail’s long-term success also relies on operational considerations, specifically, how effectively its design supports environmental sustainability and low-maintenance upkeep.

Sustainability in sensory gardens can be evaluated using criteria such as biodiversity preservation, sustainable water use, renewable energy sources and ecofriendly maintenance practices (Krzeptowska-Moszkowicz et al. 2021). For the Braille Trail, weekly maintenance is crucial to ensure safety and cleanliness, including clearing paths and maintaining signage (L. Labuscagne pers. comm., 26 November 2024). Local climate and plant growth patterns must also be considered (R. Riddles, pers. comm., 20 February 2025). While the selected plants are adapted to the region’s harsh conditions, they still require care – particularly weeding and watering during dry periods. The raised beds, designed to offer rich sensory experiences, must be replanted annually. To preserve both sensory appeal and visual order, overgrown or faded plants must be replaced as needed (T. Makola, pers. comm., 12 March 2025; L. Labuscagne pers. comm., 26 November 2024).

Interpretation boards, braille panels and pedestals are exposed to the elements and will require periodic upkeep. This includes potential repainting or resealing with protective coatings such as epoxy to maintain durability and visual quality (W. Voigt, pers. comm., 11 March 2025).

Digital tools offer real-time updates, multimedia and interactivity that can enhance accessibility and engagement. However, they must be designed with diverse users in mind, including those with limited digital literacy, and consider local circumstances such as infrastructure constraints and the broader socioeconomic context (Rivas Velarde et al. 2021:253–254). Digital components add layers of complexity, as technological change may cause interfaces and software to become outdated or incompatible. Long-term usability depends on proactive maintenance, including domain hosting renewals, platform updates and resolution of compatibility issues, all of which require ongoing investment and oversight. Sustainable planning should therefore include continuous updates of applications, software and devices to maintain a seamless and inclusive user experience.

Importantly, sustainability does not imply stasis. Content must evolve. This includes updating plantings, signage and digital media – ideally every 5 years or as needed to maintain relevance (W. Voigt, pers. comm., 11 March 2025). A clear maintenance and management plan should guide these updates to keep the trail engaging and informative (W. Voigt, pers. comm., 11 March 2025).

Lessons from the Braille Trail also highlight other critical success factors. A thorough feasibility study and detailed management plan are essential for ensuring long-term viability (W. Voigt, pers. comm., 11 March 2025). Clearly identifying and engaging a target audience from the outset allows content to be tailored to their needs, particularly when catering to smaller or specialised user groups. Ensuring that features are both accessible and meaningful is key (W. Voigt, pers. comm., 11 March 2025). Operationally, appointing a dedicated interpretation officer, supported by effective administrative structures, can streamline implementation and ongoing management (W. Voigt, pers. comm., 11 March 2025). Equally important is a genuine passion for the project, which drives commitment, innovation and sustained effort throughout planning, implementation and maintenance (T. Makola, pers. comm., 12 March 2025; R. Riddles, pers. comm., 20 February 2025; W. Voigt, pers. comm., 11 March 2025). Finally, sustained community engagement is vital. This includes involving persons with disabilities, older adults and other relevant groups throughout the design and review process to ensure the trail remains inclusive and responsive to user needs (L. Labuscagne pers. comm., 26 November 2024).

### Expert endorsement and trail reception

At the launch of the Braille Trail on 30 September 2022, Dr William Rowland – former president of the World Blind Union and long-time executive director of the South African National Council for the Blind – praised the initiative and the diversity of experiences it offers. His poem *Omdat ek die stadsrumoer* (*City Noise and I*) is featured among the garden’s site-specific poems. He stressed the importance of regular maintenance and advised the curator and staff of the Botanic Garden to remain actively involved in its upkeep (Rowland 2022). Dr Rowland also emphasised the need for consistent marketing and the ongoing involvement of blind and partially sighted advisers. He highlighted the role of organisations such as Innovation for the Blind in ensuring the trail remains accessible, meaningful and enjoyable for its intended users.

The Braille Trail was positively received in local media. The environmental television programme *50/50*, broadcast on SABC 2, featured the trail in a January 2023 segment during which visually impaired participants evaluated the experience and shared their feedback. The digital literature component also attracted international attention for its inclusive, participatory design and innovative use of technology.

Since its launch, the trail has been used by a variety of groups: learners from the Pioneer School for the Visually Impaired, school groups participating in an environmental education outreach programme and SANBI interns in Work-Integrated Learning (WIL), particularly in horticulture and fieldwork (L. Labuscagne pers. comm., 26 November 2024; T. Makola, pers. comm., 12 March 2025; W. Voigt, pers. comm., 11 March 2025). Many general visitors also start their garden visit at the trail, drawn by its accessible design and convenient location beside the parking area. The trail is also popular with older adults and families with young children. Despite this, usage by blind and visually impaired visitors remains low (L. Labuscagne pers. comm., 26 November 2024; T. Makola, pers. comm., 12 March 2025), likely because of limited public awareness and ongoing transport challenges. This highlights the need for targeted outreach, improved access and initiatives such as guided group visits.

As T. Makola (pers. comm., 12 March 2025) notes, a socially sustainable approach would involve collaborating with local disability organisations to co-plan broader use of the trail. It should also consider the many care and education services in Worcester and surrounding areas – key communities that could benefit from inclusive outdoor experiences. Promoting the trail’s inclusive features within the tourism sector could further strengthen its role as a socially embedded, accessible attraction.

## Conclusion

This article outlines the establishment of the Braille Trail in the KDNBG, South Africa, documenting a local initiative and contributing to the broader dialogue on multisensory gardens, locative literature and accessible tourism. In line with the frameworks introduced at the outset, the study demonstrates translation of the principles of universal and inclusive design, co-design and slow tourism into a botanical garden setting, offering enriched and equivalent experiences for a wide range of visitors. Situated within the Global South context, the study addresses gaps in the literature on sensory and wellbeing gardens, which have largely been shaped by perspectives from the Global North. It also highlights the potential of integrating accessible communication technologies into outdoor installations to create multisensory and locative experiences that foster place attachment and broaden participation. The implications are twofold: practically, the Braille Trail provides a model for other tourism and conservation sites seeking to align with the Convention on the Rights of Persons with Disabilities (CRPD) and the 2030 Agenda by developing universally accessible, inclusive green spaces; theoretically, the study illustrates how accessible tourism frameworks – when combined with locative literature and participatory design – can expand the discourse on sustainable tourism by linking embodied experience, technology and local identity.

Extending the application of inclusive design models in similar contexts highlights several opportunities for future development. Systematic user feedback through surveys or participatory evaluation can guide the adaptation of content, digital technologies and locative literature to diverse audiences. Kirstenbosch, Harold Porter and Walter Sisulu botanical gardens – venues that attract thousands of visitors – illustrate potential for upscaling, though careful adaptation of information and multilingual resources is needed, and funding and procedural challenges remain. Digital tools can enhance accessibility and engagement, but they must consider diverse users, local infrastructure, socioeconomic factors and technological complexity, with ongoing maintenance to ensure long-term usability. Because government funding and bureaucracy often delay implementation in South Africa, alternative approaches – such as privately funded initiatives or inclusive design in urban green spaces such as public parks – could provide more agile opportunities, with broader relevance for the Global South.

Future research could explore developing and testing educational guidelines that tailor the Braille Trail and its technologies for school programmes targeting learners with VIs. Equally important is examining sustainability – how multisensory trails can integrate local flora and fauna to support conservation and biodiversity while enhancing education and visitor wellbeing. Finally, inclusive design could be applied to other natural and heritage sites, such as accessibility-friendly educational trails in national parks, where ecological, geotourism or heritage experiences intersect with accessibility.
